# An Update on the Effects of Probiotics on Gastrointestinal Cancers

**DOI:** 10.3389/fphar.2021.680400

**Published:** 2021-12-21

**Authors:** Amirhossein Davoodvandi, Farzaneh Fallahi, Omid Reza Tamtaji, Vida Tajiknia, Zarrin Banikazemi, Hadis Fathizadeh, Mohammad Abbasi-Kolli, Michael Aschner, Maryam Ghandali, Amirhossein Sahebkar, Mohsen Taghizadeh, Hamed Mirzaei

**Affiliations:** ^1^ Student Research Committee, Kashan University of Medical Sciences, Kashan, Iran; ^2^ Network of Immunity in Infection, Malignancy and Autoimmunity (NIIMA), Universal Scientific Education and Research Network (USERN), Tehran, Iran; ^3^ Research Center for Biochemistry and Nutrition in Metabolic Diseases, Institute for Basic Sciences, Kashan University of Medical Sciences, Kashan, Iran; ^4^ Students’ Scientific Research Center, Tehran University of Medical Sciences, Tehran, Iran; ^5^ Department of Surgery, School of Medicine, Iran University of Medical Sciences, Tehran, Iran; ^6^ Department of Laboratory Sciences, Sirjan Faculty of Medicine Sciences, Sirjan, Iran; ^7^ Department of Medical Genetics, Faculty of Medical Sciences, Tarbiat Modares University, Tehran, Iran; ^8^ Department of Molecular Pharmacology, Albert Einstein College of Medicine, Bronx, NY, United States; ^9^ School of Medicine, Iran University of Medical Sciences, Tehran, Iran; ^10^ Applied Biomedical Research Center, Mashhad University of Medical Sciences, Mashhad, Iran; ^11^ Biotechnology Research Center, Pharmaceutical Technology Institute, Mashhad University of Medical Sciences, Mashhad, Iran

**Keywords:** probiotic, gastrointestinal disorders, cancer, pathology, therapy

## Abstract

Because of their increasing prevalence, gastrointestinal (GI) cancers are regarded as an important global health challenge. Microorganisms residing in the human GI tract, termed gut microbiota, encompass a large number of living organisms. The role of the gut in the regulation of the gut-mediated immune responses, metabolism, absorption of micro- and macro-nutrients and essential vitamins, and short-chain fatty acid production, and resistance to pathogens has been extensively investigated. In the past few decades, it has been shown that microbiota imbalance is associated with the susceptibility to various chronic disorders, such as obesity, irritable bowel syndrome, inflammatory bowel disease, asthma, rheumatoid arthritis, psychiatric disorders, and various types of cancer. Emerging evidence has shown that oral administration of various strains of probiotics can protect against cancer development. Furthermore, clinical investigations suggest that probiotic administration in cancer patients decreases the incidence of postoperative inflammation. The present review addresses the efficacy and underlying mechanisms of action of probiotics against GI cancers. The safety of the most commercial probiotic strains has been confirmed, and therefore these strains can be used as adjuvant or neo-adjuvant treatments for cancer prevention and improving the efficacy of therapeutic strategies. Nevertheless, well-designed clinical studies are still needed for a better understanding of the properties and mechanisms of action of probiotic strains in mitigating GI cancer development.

## Introduction

The incidence of gastrointestinal (GI) neoplasms is rapidly increasing globally (Ashrafizadeh et al., 2020; Pourhanifeh et al., 2020; Shafabakhsh et al., 2021). GI cancers are a complex set of heterogenous diseases and disorders (Wang et al., 2021) and are classified into more frequent sporadic and rare inherited forms. Environmental and genetic risk factors can cooperatively alter normal tissue into a precursor or a premalignant injury, culminating in malignancy. While the precise genetic mechanisms are somewhat understood in a tissue-type– and cell-type–specific context, many common aspects exist between GI cancers of heterogenous origin (Wang et al., 2021). Consistent with the advances made in developing new diagnostic and therapeutic approaches for GI cancers, several probiotic strains are being used as nutritional supplements.

Probiotics are a group of viable microorganisms including bacteria and yeasts that if consumed in sufficient amounts, may afford health benefits to the host (Ganguly et al., 2011; Tamtaji et al., 2019a; Tamtaji et al., 2019b; Alipour Nosrani et al., 2021; Davoodvandi et al., 2021). The major advantage of probiotic administration is its ability to maintain gut microbial homeostasis, reduce pathogenic microorganisms in the GI tract, and restores homeostasis of intestinal microorganisms (Floch et al., 2011; Butt and Epplein, 2019). Moreover, by modulating microbiota and immune responses, decreasing bacterial translocation, promoting the function of the gut barrier, inducing anti-inflammatory properties, triggering anti-pathogenic activity, and decreasing tumor development and metastasis, probiotics might contribute to the prevention and treatment of GI cancers (Servin, 2004; Cotter et al., 2005; Javanmard et al., 2018). Considering the potential roles of *Helicobacter pylori* (H. pylori) in the initiation of colorectal (Teimoorian et al., 2018; Butt and Epplein, 2019) and gastric cancers (Alfarouk et al., 2019), the possible properties of probiotics against GI neoplasm in humans have been investigated in relation to their suppressive effects on H. pylori (Taremi et al., 2005; Sanders et al., 2013; Russo et al., 2014; Khoder et al., 2016; Rasouli et al., 2017). By triggering immune activity, probiotics, as functional dietary supplements, may mitigate neoplastic predisposition and development of GI cancers (Liong, 2008; Zuccotti et al., 2008; Kumar et al., 2010; De Preter et al., 2011; Zhang et al., 2011).

## Clinical Overview on GI Neoplasms

Carcinogenesis is a multistage process characterized by genetic mutations (Nowell, 1976; Yuasa, 2003; Vogelstein and Kinzler, 2004). In the past, initiation and progression of tumors were considered as distinct processes. A critical observation that led to the multistage hypothesis was that neoplasm was clonal, with each neoplastic cell originating from a single progenitor (Nowell, 1976; Cahill et al., 1999). This model implied that genetic mutations required for neoplastic transformation did not occur at once, but rather progressively. With each stage in this process, the transforming cell obtained a new mutation that promoted cell survival or proliferation.

A cell clone was developed with all of the necessary aspects for neoplastic transformation through evolution or natural selection. Selection is a critical element of this process because mutations are random events; thus, only rare mutations result in activation of cell survival and growth-promoting pathways or inactivation of apoptotic pathways or tumor suppressors (Ponder, 2001). These mutations impart a selective survival and growth dominance to that cell and its progeny. This leads to the expansion of that cell into a clonal population. Further mutations that occur in cells of that clonal population provide a few rare cells with new superiority. These daughter cells are subjected to an additional round of clonal expansion. This process continues, building on round after round of clonal expansion, till a mass is generated, and neoplastic transformation has taken place (Nowell, 1976; Cahill et al., 1999).

The specific number of somatically acquired gene mutations necessary for neoplastic transformation is dependent upon which genes and tissues are targeted. In common solid tumors, such as those derived from the colon or pancreas, an average of 33–66 genes displays subtle somatic mutations that would be expected to alter their protein products. About 95% of these mutations are single-base substitutions (such as C > G), whereas the remainder are deletions or insertions of one or a few bases (such as CTT > CT). Of the base substitutions, 90.7% result in missense changes, 7.6% result in nonsense changes, and 1.7% result in alterations of splice sites or untranslated regions immediately adjacent to the start and stop codons (Vogelstein et al., 2013).

Typically, benign dysplastic intermediates develop before GI neoplasm. Indeed, they do not originate from normal tissues directly, and the dysplastic lesions are characterized by their morphology and categorized based on certain pathological indicators (Said, 2012). For example, in the colon, the adenoma–carcinoma pattern shows this promotion from normal mucosa to invasive carcinoma *via* dysplastic intermediates. This pattern has been well supported by many pathological and animal studies (Kim and Lance, 1997; Lynch and Hoops, 2002).

The same multistep pattern from normal tissue via dysplastic intermediates to malignancy has been shown for human pancreatic, esophageal, and gastric cancers (Hruban et al., 2001; Yuasa, 2003; Hruban et al., 2004; Lin and Beerm, 2004). Cancer always emerges in a dysplastic precursor lesion that is histologically or grossly apparent. Current models have shown that the sequence of events prior to intestinal gastric cancer is as follows: atrophic gastritis, intestinal-metaplasia, and adenomas, which develop into carcinomas (Yuasa, 2003). Precursor lesions that lead to pancreatic cancer have been formally agreed upon, and the characteristics necessary for their classification have been established (Hruban et al., 2001; Hruban et al., 2004). These criteria classify the pancreatic lesions for both scientific and clinical uses.

The concept of cancer stem cells highlighted new perspectives in understanding this disease. Although it is tempting to explain tumor formation and metastasis by the presence of stem cells, after almost a decade of intense research, it seems that cancer stem cells fail to explain how neoplasia evolves. It seems most likely that this population of cells is not a defined group of cells resting in a niche and populating the tumor with amplifying cells, but rather, that few or maybe multiple cells within the tumor can function as cancer stem cells if induced, yet also revert to the state of a “normal” cancer cell. In general, cancer stem cells resulting from mutations in stem/progenitor cells most likely undergo uncontrolled proliferation (Li and Neaves, 2006; Abdul Khalek et al., 2010; Welte et al., 2010).

## Probiotic and Cancer Therapy

Advances have been made over the last century to develop anticancer drugs that lead to drastically reducing of the side effects of medications (Falzone et al., 2018). However, the beneficial effects of probiotics on metabolic profiles and biomarkers of inflammation and oxidative stress were previously reported (Asemi et al., 2012a; Asemi et al., 2012b; Tajadadi-Ebrahimi et al., 2014; Bahmani et al., 2016). Modifying the intestinal microbiome with oral probiotics has been applied to decrease side effects associated with drugs. The adverse effects caused by anticancer treatments mainly include mucositis and diarrhea. Among the advantages of probiotics are their low cost and general safety (Rondanelli et al., 2017). Probiotic application in clinical practice has displayed a wide range of advantages, such as improving antibiotics and *Clostridium difficile*-related diarrhea and respiratory tract infections (Rondanelli et al., 2017). Populating the gut microbiota in cancer patients with probiotics re-establishes both the functionality and quantities of commensal bacteria, which are reduced after treatments (Zitvogel et al., 2018). Nonetheless, probiotic administration in several clinical trials has been shown to re-establish healthy intestinal microbiota composition and to diminish diarrhea and other treatment-related damages to the gut, such as mucositis (Mego et al., 2013). Consistently, *Lactobacillus* containing probiotics prevent diarrhea and mucositis in individuals, who received chemotherapy/radiotherapy for pelvic malignancy (Gianotti et al., 2010; Lalla et al., 2014).

The specific mechanism associated with the antitumor properties of probiotics remains unclear. Gut microbiota affect a variety of pathways, which are considered to play a central role in this process. Primarily, probiotic bacteria play an essential role in the preservation of homeostasis, thus maintaining sustainable physicochemical conditions in the colon. Reduced pH causing inter alia by the excessive presence of bile acids in feces may be a direct cytotoxic factor affecting colonic epithelium leading to colon carcinogenesis. Regarding their involvement in the modulation of the pH and bile acid profile, probiotic bacteria, such as L. acidophilus and B. bifidum, have shown efficacy in cancer prevention (Biasco et al., 1991; Bernstein et al., 2005; Jia et al., 2018).

Probiotic strains are also responsible for maintaining the balance between the quantity of other participants of natural intestinal microflora and their metabolic activity. Putrefactive bacteria, such as *Escherichia coli* and *Clostridium perfringens*, naturally present in the gut, have been proven to be involved in production of carcinogenic compounds using enzymes such as β-glucuronidase, azoreductase, and nitroreductase (Górska et al., 2019).

Another cancer-preventing strategy involving probiotic bacteria, such as chiefly *Lactobacillus* and *Bifidobacillus* strains, has been linked to the binding and degradation of potential carcinogens. Mutagenic compounds associated with the increased risk of colon cancer are commonly found in unhealthy food, especially fried meat. Ingestion of the *Lactobacillus* strain by human volunteers alleviated the mutagenic effect of diet rich in cooked meat, which resulted in decreased urinary and fecal excretion of heterocyclic aromatic amines (HAAs) (Lidbeck et al., 1992; Hayatsu and Hayatsu, 1993; Górska et al., 2019).

Many beneficial compounds produced and metabolized by gut microbiota have been demonstrated to play an essential role in maintaining homeostasis and suppressing carcinogenesis. A specific population of gut microbiota is dedicated to the production of short-chain fatty acids (SCFAs) such as acetate, propionate, and butyrate as a result of the fermentation of fiber-rich prebiotics. Except for their principal function as an energy source, SCFAs have also been proven to act as signaling molecules affecting the immune system, cell death, and proliferation as well as intestinal hormone production and lipogenesis, which explains their crucial role in epithelial integrity maintenance (Garrett, 2015; Requena et al., 2018; Górska et al., 2019).

**FIGURE 1:**
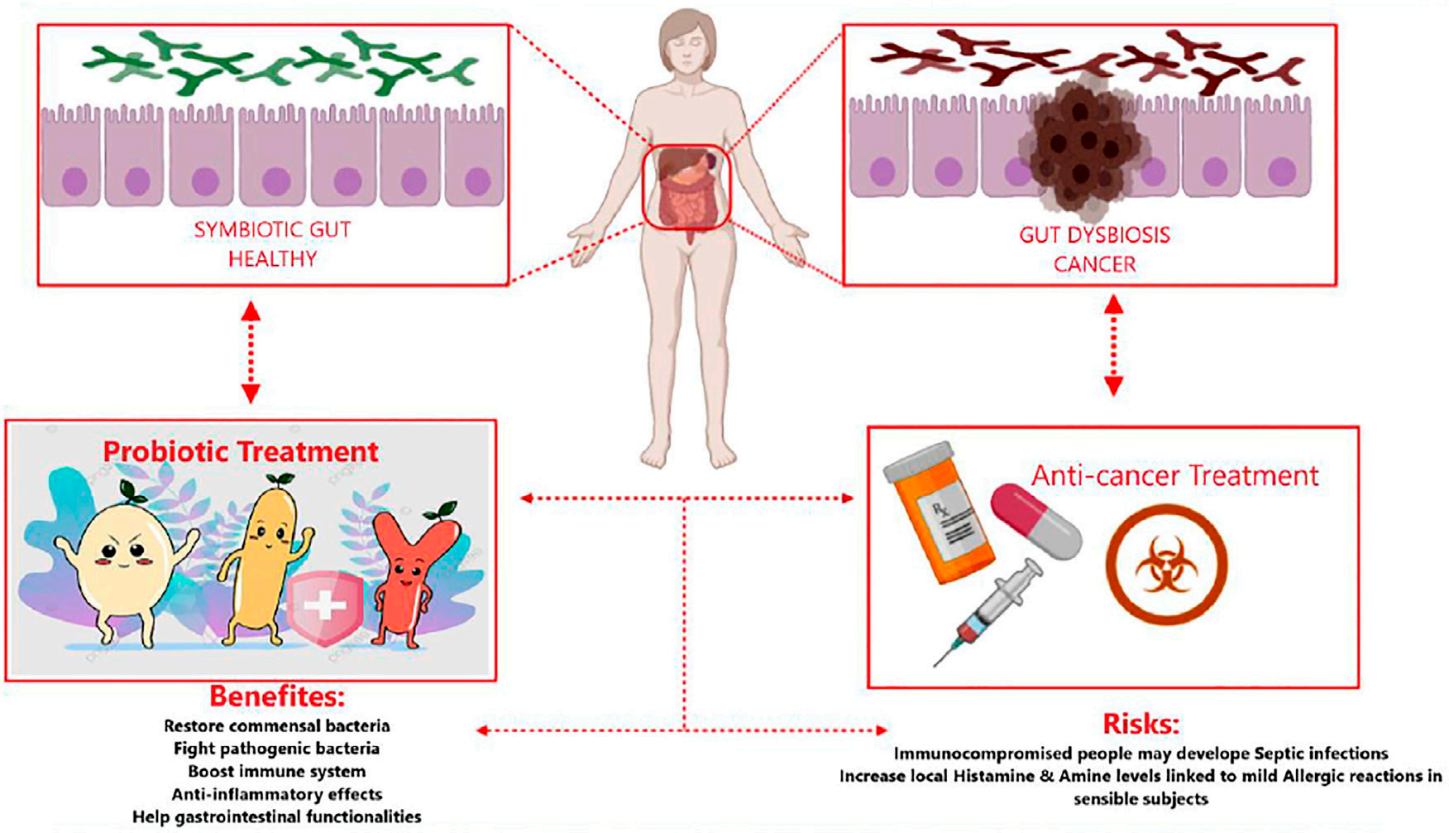
Risks and benefits of probiotics associated with cancer treatment. Schematic depiction of healthy gut microbiota in humans, occupied by symbiotic bacteria **(top left box)** against tumor-affected microbiota and dysbiosis of the gut **(top right box)**. Anticancer treatment may negatively influence gut microbiota, leading to dysbiotic unbalance **(bottom right square)**. Probiotic administration may re-adjust the dysbiotic conditions mediated by tumor growth and treatment. Probiotics may improve gastrointestinal therapy–related side effects, so they re-establish the intestinal symbiosis **(bottom left square)**. The application of probiotics in anticancer therapy has benefits and risks **(central bottom box)**.

Figure 1 shows both the advantages and potential disadvantages of probiotic administration as adjuvants during cancer treatments. The figure highlights show probiotics’ regulation of the gut’s subtle equilibrium, from microbial imbalance (dysbiotic) to functional and healthy microbiota.

## Effects of Probiotics on Gastrointestinal Cancer Cells

### Probiotics and Gastric Cancer

*H. pylori*-mediated inflammation is one of the potential factors in the induction of gastric cancer in infected populations (Moss, 2017). Evidence evaluating the anti-gastric cancer effects of probiotics has focused on *H. pylori*-induced pathophysiology of this type of cancer. Maleki-Kakelar and others reported that by mediating numerous molecular pathways, *Lactobacillus plantarum* (*L. plantarum*) caused significant inhibitory effects on the *H. pylori* growth rate. Upon downregulation of the AKT gene and upregulation of the phosphatase and tensin homolog (PTEN), Bcl-2–associated X (Bax), and toll-like receptor 4 (TLR4), *L. plantarum* significantly inhibited the proliferation of AGS and CRL-1739 human gastric cell lines (Maleki-Kakelar et al., 2020). Interleukin-8 (IL-8) is an inflammatory chemokine that plays critical roles in inflammatory pathways (Meniailo et al., 2018). In the human gastric epithelial cell line-1 (GES-1), *Lactobacillus bulgaricus* (*L. bulgaricus*) inhibited the production of IL-8. In addition, *Lactobacillus acidophilus* (*L. acidophilus*) and *L. bulgaricus* inhibited adhesion of *H. Pylori* to GES-1 cells that attenuated inflammation in these cells (Song et al., 2019). Lin et al. reported that supplementation with *Lactobacillus fermentum* P2 (*L. bacillus* P2), *L. casei* L21, *L. rhamnosus* JB3, or their combination in *H. pylori*-infected mice reduced the expression level of interferon gamma (IFN-γ) along with interleukin-1 beta (IL-1β). Besides, *H. pylori* concentrations in the stomach of infected mice were decreased after probiotic supplementation (Lin et al., 2020). Another study demonstrated that *L. acidophilus*, *L. plantarum*, and *L. rhamnosus* supplementation significantly attenuated *H. pylori*-induced inflammation *in vivo* (Asgari et al., 2020). As mentioned earlier, most of the anti-gastric cancer studies have been directed at the inhibitory effects on *H. pylori* infection. Further experimental studies are needed for evaluating the effects of probiotic on gastric cancer inhibition mechanistically.

Ornithine decarboxylase is a crucial enzyme in the polyamine biosynthesis pathway and is responsible for catalyzing the decarboxylation of ornithine into putrescine (Svensson et al., 2008). Ornithine decarboxylase is a neovascularization agent in tumoral cells and has been overexpressed in tumors of epithelial origin including colorectal, prostate, and gastric cancers (Ma et al., 2007). Russo and others demonstrated that treatment with *L. rhamnosus* GG homogenate and cytoplasm extracts significantly decreased the activity of ornithine decarboxylase, reducing the polyamine content of HGC-27 human gastric cancer cells. Furthermore, in comparison with the untreated control group, probiotic treatment considerably increased the ratio of Bax/Bcl-2 (Russo et al., 2007). Xie and others reported that 8-day postoperative probiotic supplementation in gastric cancer patients significantly reduced diarrhea occurrence. Furthermore, in probiotic-induced patients, the expression level of interleukin-6 (IL-6), interleukin-8 (IL-8), and tumor necrosis factor alpha (TNF-α) was significantly decreased compared with that in patients in the control group (Xie et al., 2018).

The urokinase plasminogen activator (uPA) is an important serine proteinase enzyme which catalyzes the production of active protease plasmin from its proenzyme plasminogen (Pavón et al., 2016; Mahmood et al., 2018). The activation of the uPA is dependent on the expression of the uPA receptor (uPAR) in the cell surface. By increasing the activity of matrix metalloproteinases (MMPs), plasmin degrades extracellular matrix components, contributing to cancer metastasis and invasion (Beamish et al., 2019). Therefore, the activation of the uPA/uPAR system plays crucial roles in the induction of invasiveness and metastatic features in cancerous cells (Rubina et al., 2017). Rasouli et al. reported that treatment with *Lactobacillus reuteri* (*L.reuteri*), in AGS gastric cancer cells, downregulated the expression level of the uPA/uPAR gene (Rasouli et al., 2017). Table 1 provides a summary of studies on probiotics and gastric cancers. Nami et al. studied the anticancer effects of *Lactobacillus plantarum* species on human cancer cell lines (cervical, HeLa; gastric, AGS; colon, HT-29; and breast, MCF-7) and on a human normal cell line (HUVEC). The strain exhibited desirable probiotic properties and anticancer activity against the tested human cancer cell lines; no significant cytotoxic effects on normal cells were exhibited (Nami et al., 2014).

**TABLE 1:** Probiotics and gastric cancer.

Cancer cell line	Probiotic agent	Probiotic concentration	Duration of the study	Effect (s)	Model	Sample (n)	Ref.
AGS	*Lactobacillus reuteri*	1.5 × 10^8^ CFU/ml	24, 48, and 72 h	Inhibited cell proliferation and decreased uPA and uPAR	*In vitro*	NA	Rasouli et al. (2017)
HGC-27	*Lactobacillus paracasei* IMPC2.1 and *Lactobacillus rhamnosus* GG	1 × 10^8^ CFU/ml	24 or 48 h	Induced apoptosis and inhibited tumor growth	*In vitro*	NA	Orlando et al. (2012)
NCI-N87 and AGS	*Lactobacillus acidophilus* 74-2 and *Bifidobacterium lactis* 420	8.24 × 10^7^ and 2.20 × 10^8^ CFU, respectively	NA	Upregulated the expression of COX-1	*In vitro*	NA	Mahkonen et al. (2008)
HGC-27	*Lactobacillus rhamnosus GG* (ATCC 53103)	1 × 10^8^ CFU/ml	24 and 48 h	Reduced the polyamine content and neoplastic proliferation	*In vitro*	NA	Linsalata et al. (2010)
HGT-1	*Propionibacterium freudenreichii* ITG P9	9 × 10^12^ CFU/ml	24, 48, or 72 h	Induced caspase activation and cytochrome *c* release	*In vitro*	NA	Cousin et al. (2012)
AGS	*Lactobacillus fermentum* UCO-979C and *Lactobacillus casei* Shirota	1.5 × 10^9^ CFU/ml	0–48 h	Inhibited urease activity of *H. pylori*	*In vitro*	NA	Salas-Jara et al. (2016)
AGS	*Lactobacillus plantarum* 5BL	NA	12, 24, and 48 h	Induced anti-proliferative effects and apoptosis	*In vitro*	NA	Nami et al. (2014)
Postoperative patients with gastric cancer	NA	NA	7–8 days	Decreased the expression of IL-6, IL-8, and TNF-α	Human	70	Xie et al. (2018)
Gastric cancer patients	*Bifidobacterium*	NA	4 weeks	Decreased SIBO and symptoms of gastric cancer in the intervention group	Human	112	Liang et al. (2016)

uPA, urokinase-type plasminogen activator; uPAR, urokinase-type plasminogen activator receptor; COX-1, cyclooxygenase 1; *H. pylori*, *Helicobacter pylori*; IL-6, interleukin 6; IL-8, interleukin 8; TNF-α, tumor necrosis factor alpha; SIBO, small intestine bacterial overgrowth.

### Probiotics and Colon Cancer

#### Probiotics and Colon Cancer in Human Studies

One of the important goals in treating colorectal cancer patients is improving their quality of life. The role of probiotics in decreasing the symptoms and improving the quality of life in colorectal cancer patients has been evaluated at different stages of the disease. Lacidofil supplementation for 12 weeks in patients with colorectal cancer reduced the frequency of bowel symptoms while promoted functional well-being scores compared with those of patients in the placebo group (Lee et al., 2014). Zonulin (haptoglobin 2 precursor) is a regulator of tight junctions and intestinal permeability in the wall of the digestive tract (Sturgeon and Fasano, 2016). The increased serum level of zonulin was associated with autoimmunity, inflammatory diseases, and gastrointestinal cancers (Mörkl et al., 2018). Supplementation for 16 days (6 days preoperatively and 10 days postoperatively) with an admixture of *L. plantarum*, *L. acidophilus*-11, and *B. longum*-88 in colorectal cancer patients caused significant reduction in serum concentrations of zonulin as well as the duration of postoperative pyrexia, antibiotic therapy, and infectious complications in comparison with those in the placebo group. In addition, probiotic intervention inhibited the p38 mitogen-activated protein kinase signaling pathway (Liu et al., 2012). Yang and others reported that probiotic intervention with an admixture of *B. longum*, *L. acidophilus*, and *Enterococcus faecalis (E. faecalis)* for 12 days (5 days preoperatively and 7 days postoperatively) reduced the number of days to first defecation, days to first flatus, and diarrhea in the probiotic-treated group (Yang et al., 2016). 5-Fluorouracil (5-FU) is one of the most effective drugs for chemotherapy in colorectal cancer patients (Fu et al., 2019). However, its use is associated with diarrhea (Cheng et al., 2020). In a recent study, 24-week supplementation with *L. rhamnosus* GG in colorectal cancer patients who received 5-FU, the frequency of diarrhea was significantly decreased (Osterlund et al., 2007). Aisu et al. reported that supplementation with a probiotic mixture containing *Enterococcus faecalis* T110, *Clostridium butyricum* TO-A, and *Bacillus mesentericus* TO-A in colorectal cancer patients (*n* = 75) significantly diminished the occurrence of superficial incisional infection compared with that in untreated patients (Aisu et al., 2015). *Fusobacterium* is an important bacterial pathogen, which causes overexpression of E-cadherin/β-catenin and subsequent colorectal cancer proliferation (Zhou et al., 2018). Using an admixture of *B. longum*, *L. acidophilus,* and *Enterococcus faecalis* (*E. faecalis*) in colorectal cancer patients (*n* = 11) for 5 days significantly altered mucosa-associated microbiota of the intestine. Furthermore, probiotic intervention reduced the secretion of taxon assigned to the *Fusobacterium* (Gao et al., 2015). The treatment of colorectal cancer patients (*n* = 84) with a combination of probiotics, which consisted of *L. acidophilus*, *L. plantarum*, *B. lactis*, and *Saccharomyces boulardii* (1 day preoperatively and 15 days postoperatively), significantly decreased pneumonia, surgical site infections, anastomosis leakage, and need for mechanical ventilation compared with those who did not receive probiotic supplementation (Kotzampassi et al., 2015). Other studies evaluating the properties of probiotics in colorectal cancer patients are summarized in Table 2.

**TABLE 2:** Probiotics and colon cancer in human studies.

Subject	Probiotic agent	Probiotic concentration	Duration of the study	Effect (s)	Sample (n)	Ref.
Postoperative patients with colorectal cancer	*Lactobacillus acidophilus* LA-5*, Lactobacillus plantarum, Bifidobacterium lactis* BB-12*,* and *Saccharomyces boulardii*	1.75 × 10^9^, 0.5 × 10^9^,1.75 × 10^9^, and 1.75 × 10^9^ CFU per capsule, respectively	16 days (1 day prior to operation and 15 days after operation)	Modulated the gene expression of SOCS3 and significantly decreased postoperative complications including mechanical ventilation, infections, and anastomotic leakage	84	Kotzampassi et al. (2015)
Colorectal cancer	*Bifidobacterium lactis*	1 × 10^9^ CFU/gr	4 weeks	The amounts of IL-1β, IL-2, IL-12, and hs-CRP in the probiotic group was significantly lower than those in symbiotic and prebiotic intervention groups	19	Worthley et al. (2009)
Perioperative patients with colorectal cancer	*Bifidobacterium longum* (BB536) and *Lactobacillus johnsonii* (La1)	2 × 10^7^ CFU/d and 2 × 10^9^ CFU/d (two separate doses)	8 days (3 days before operation and 5 days after operation)	The count of CD3, CD4, and CD8 in both of the intervention groups was greater than that in the placebo group	11 and 10 (two groups)	Gianotti et al. (2010)
Perioperative patients with colon cancer	*Bifidobacterium bifidum*	1 × 10^10^ CFU	17 days (7 days before operation and 10 days after operation)	Surgical site infection in the probiotic group significantly decreased compared to that in the antibiotic group	100	Sadahiro et al. (2014)
Colorectal cancer	*Bifidobacterium*	NA	4 weeks	Decreased the symptoms of colorectal cancer in the intervention group	88	Liang et al. (2016)
Colorectal cancer	*Lactobacillus rhamnosus* R0011 and *Lactobacillus acidophilus* R0052	2 × 10^9^ CFU	12 weeks	Attenuated bowel symptoms and improved quality of life in colorectal cancer subjects	28	Lee et al. (2014)
Perioperative patients with colorectal and colon cancer	*Bacillus natto* and *Lactobacillus acidophilus*	NA	3 months	In the colonic group, defecation frequency, anal pain, and the Wexner score were significantly better than those in patients in the rectal cancer group	77	Ohigashi et al. (2011)
Perioperative patients with colorectal cancer	*Enterococcus faecalis* T110*, Clostridium butyricum* TO-A*,* and *Bacillus mesentericus* TO-A	2 mg, 2 mg, and 10 mg, respectively, per each tablet	6–30 days (3–15 days prior to and after the surgery)	Enhanced the immune responses and improved the intestinal microbial environment in the probiotic group	75	Aisu et al. (2015)
Healthy subjects	*Bifidobacterium longum* (BB536-y)	NA	5 weeks	Inhibited colorectal carcinogenesis	14	Ohara and Suzutani, (2018)
Colorectal cancer	*Lactobacillus acidophilus* and *Lactobacillus plantarum*	NA	NA	Reduced the severity of colorectal cancer	25	Zinatizadeh et al. (2018)
Perioperative patients with colorectal cancer	*Bifidobacterium longum, Lactobacillus acidophilus,* and *Enterococcus faecalis*	0.21 gr (1 × 10^8^ CFU/gr) in each capsule	3 days before operation	Promoted the expression levels of IgG and sIgA, while diminished the IL-6 and CRP serum in the intervention group	30	Zhang et al. (2012)
Perioperative patients with colorectal cancer	*Lactobacillus acidophilus, Lactobacillus casei, Lactobacillus lactis, Bifidobacterium bifidum, Bifidobacterium longum,* and *Bifidobacterium infantis*	3 × 10^10^ CFU	7 days before operation	Hospital stay duration in the probiotic-administrated patients was shorter than that of the patients in the placebo group	20	Tan et al. (2016)
Colorectal cancer	*Bifidobacterium longum, Lactobacillus acidophilus,* and *Enterococcus faecalis*	6 × 10^7^ CFU	5 days	Probiotic treatment altered the mucosal microbial flora	11	Gao et al. (2015)
Perioperative patients with colorectal cancer	*Lactobacillus plantarum, Lactobacillus acidophilus,* and *Bifidobacterium longum*	2 g⁄day in a concentration of 2.6 × 10^14^ CFU	16 days (6 days preoperatively and 10 days postoperatively)	Probiotic treatment upregulated the mucosal tight junction protein expression	50	Liu et al. (2011a)
Patients with colorectal tumors	*Lactobacillus casei* Shirota	1 × 10^10^ CFU/gr	4 years	Occurrence of tumors much significantly decreased in probiotic-administrated subjects compared with that in other groups	99	Ishikawa et al. (2005)
Perioperative patients with colorectal cancer	*Bifidobacterium longum, Lactobacillus acidophilus,* and *Enterococcus faecalis*	≥3 × 10^7^ CFU/gr	12 days (5 days preoperatively and 7 days postoperatively)	The incidence of diarrhea in the probiotic group was lower than that in the placebo group	30	Yang et al. (2016)
Perioperative patients with colorectal cancer	*Lactobacillus plantarum, Lactobacillus acidophilus 11,*and *Bifidobacterium longum 88*	2.6 × 10^14^ CFU	16 days (6 days preoperatively and 10 days postoperatively)	Treatment with the probiotic decreased the infection rate, serum zonulin concentration, and duration of antibiotic therapy	75	Liu et al. (2012)
Healthy subjects	*Lactobacillus rhamnosus LC705* and *Propionibacterium freudenreichii ssp. shermanii JS*	4 × 10^10^ CFU (2 × 10^10^ CFU of each strain per day)	4 weeks	Probiotic supplementation decreased the activity of β-glucosidase	37	Hatakka et al. (2008)

SOCS3 suppressor of cytokine signaling 3; IL-1β, interleukin 1 beta; IL-2, interleukin 2; IL-12, interleukin 12; hs-CRP, high-sensitivity C-reactive protein; IgG, immunoglobulin G; sIgA, sensitive immunoglobulin A; CRP, C-reactive protein.

#### Probiotics and Colon Cancer in Animal Studies

**FIGURE 2:**
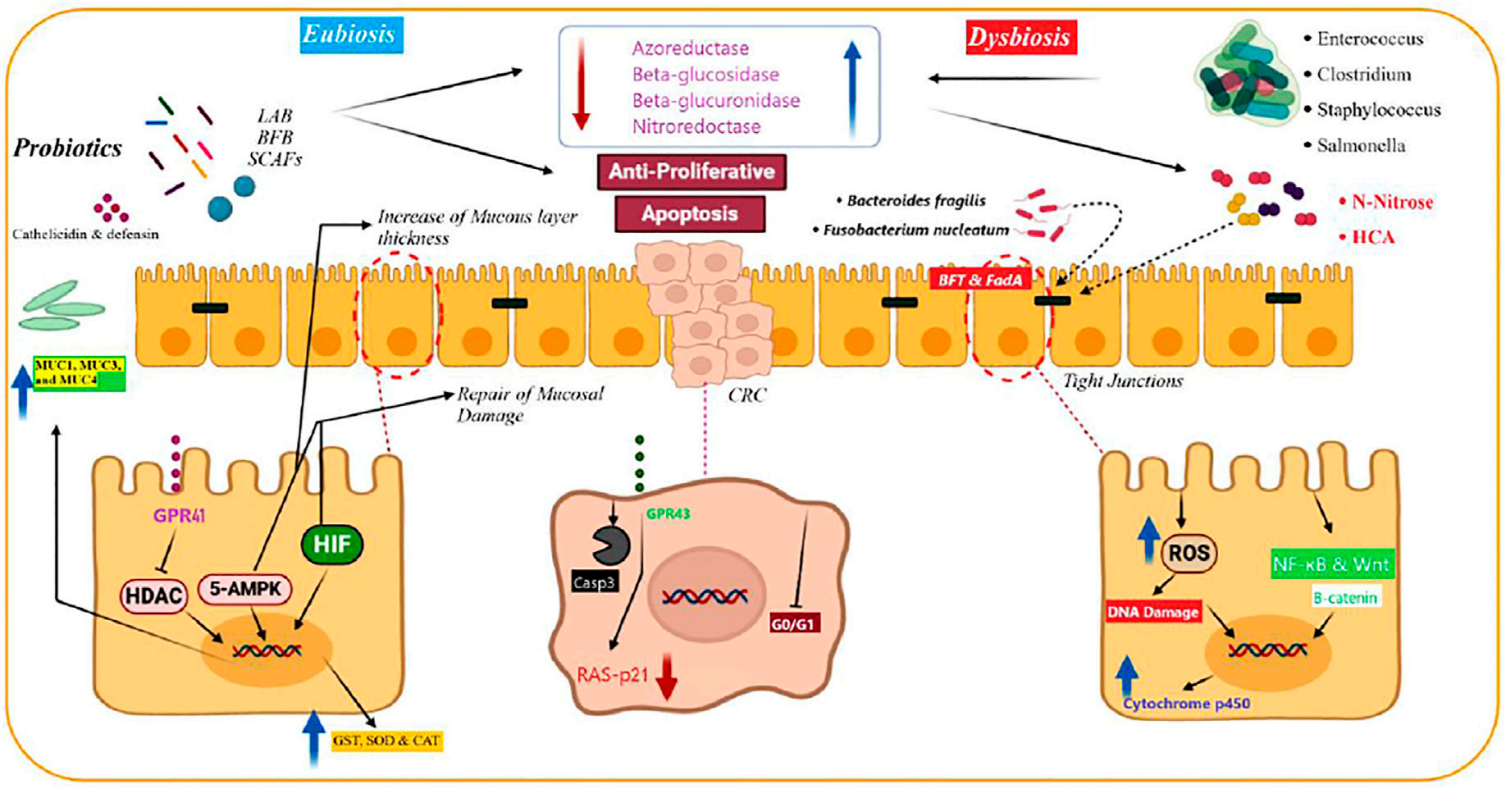
Physiological nonspecific mechanisms of probiotics for preventing and treating colorectal cancer (CRC). Probiotics produce short‐chain fatty acid (SCFA) and mediate apoptotic and anti-proliferative reactions in CRC cells. Produced SCAFs by probiotics protect the intestinal tract by preventing the histone deacetylases (HDACs) and overexpression of mucins, including MUC1, MUC3, and MUC4. SCFAs activate 5′‐adenosine monophosphate‐activated protein kinase. This is a critical factor in keeping the hypoxia‐inducible factor *via* SCAFs, which improves the epithelial duct’s survival and function. Probiotics elevate antimicrobial peptides, including defensin and (LL‐37) cathelicidin, from the intestinal mucosal layer. These peptides protect them against bacterial inoculation and invasion. Probiotics inhibit enzymatic activity of pathogenic bacteria, including enzymes such as nitroreductase, β‐glucuronidase, azoreductase, and β‐glucosidase. They also decrease the production of carcinogenic agents. Probiotics inhibit carcinogenic agents (N‐nitrous and heterocyclic aromatic amines [HCA]) by two mechanisms (deactivation and binding). They are potent mutagens and result in carcinogenic mutations in intestinal cells. Moreover, probiotics increase the antioxidant enzyme production and inactivate carcinogen‐deactivating agents, including glutathione reductase, glutathione‐S‐transferase (GST), superoxide dismutase (SOD), glutathione peroxidase, and catalase (CAT), and decrease their adverse effects. Besides, probiotics eliminate the risk of CRC development due to metabolites that have effects on the cytochrome p450. This figure is adapted from Eslami et al., (2019).

Probiotics exert their effects via activation or inhibition of cellular and molecular pathways (Figure 2). TNF-α is a pro-inflammatory cytokine which is produced by macrophages and T-cells and has numerous immunological roles in the regulation of inflammation (Farajzadeh et al., 2017). Mi et al. reported that chemotherapy induced significant increases in the levels of interleukin-6 (IL-6), interleukin-1 beta (IL-1β), and TNF-α expression in rats. In turn, treatment with *Bifidobacterium infantis* (*B. infantis*) decreased the level of the aforementioned cytokines. Furthermore, probiotic treatment reduced the expression of cytokines related to Th17 and Th1 cells, and these changes led to decreased chemotherapy-induced mucositis (Mi et al., 2017). Ras-p21 is an oncoprotein and plays critical roles in the induction of different cancers (Banys-Paluchowski et al., 2018). In rats with azoxymethane-induced colorectal cancer, *Bifidobacterium longum* (*B. longum*) administration significantly suppressed the tumor volume, tumor incidence, cell proliferation, and the expression of ras-p21 (Singh et al., 1997). Administration of *L. plantarum* and *L. rhamnosus* promoted the expression of anti-oxidant enzymes such as glutathione, superoxide dismutase, catalase, glutathione reductase, glutathione peroxidase, and glutathione-S-transferase in rats with 1,2-dimethylhydrazine-induced colorectal cancer. Furthermore, the treatment increased the concentrations of pro-apoptotic agents, such as p53, B-cell lymphoma 2 (Bcl-2), BCL2-associated X (Bax), caspase-9, and caspase-3, which are involved in the p53-mediated apoptotic pathway (Walia et al., 2018). Walia and others demonstrated that 16-week supplementation with *L. plantarum* and *L. rhamnosus* decreased the expression of cyclooxygenase-2 (COX-2). Therefore, it appears that suppressing COX-2 is a potential protective mechanism against colon cancer development, leading to decreased tumor volume and incidence (Walia et al., 2015). Ki-67 is a tumor proliferative marker that is associated with the upper proliferation rate in various types of cancers (Sun and Kaufman, 2018). An admixture of *L. fermentum* and *L. acidophilus* in the mouse model of colorectal cancer reduced tumor growth, survival, and proliferation and decreased the expression of Ki-67 compared with those of the placebo group. Concomitantly, probiotic supplementation had no significant effects on the expression of cleaved caspase-3, E-cadherin, and β-catenin in comparison with that of the other group (Kahouli et al., 2017). In the dimethylhydrazine-induced colon cancer model, the probiotic strain *L. rhamnosus* GG suppressed the expression of β-catenin, COX-2, and TNF-α. Moreover, probiotic supplementation upregulated the expression of pro-apoptotic proteins Bax, p53, and caspase 3 and downregulated the expression of Bcl-2 as an anti-apoptotic agent (Kumar et al., 2012). Agah and others compared the efficacy of *L. acidophilus* and *B. bifidum* probiotic strains against the azoxymethane-induced mouse model of colon cancer. The results showed that the colonic lesions incidence was decreased after probiotic intervention compared with that of the control group, and these effects were more potent for *L. acidophilus* than for *B. bifidum*. Serum concentrations of tumor markers CEA and CA19-9 were reduced after treatment with probiotics, while the expression of interferon gamma (IFN-γ), interleukin-10(IL-10), and the count of CD4^+^ and CD8^+^ cells were upregulated upon intervention (Agah et al., 2019a). Wang et al. evaluated the efficacy of 12-week probiotic VSL#3 supplementation on azoxymethane/dextran sulfate sodium-induced colitis-associated carcinogenesis (1.5 × 10^9^ CFU). Compared with that of the untreated group, probiotic supplementation downregulated the expression level of IL-6 and TNF-α in a considerable manner. Furthermore, probiotic intervention decreased the *Oscillibacter* and *Lachnoclostridium* genera, coupled with increased presence of *Bacillus* and *Lactococcus* genera in the fecal microbial composition of mice samples (Wang et al., 2018a). The c-Jun NH_2_-terminal kinase (JNK) is a major protein kinase which belongs to the MAPK signaling pathway and plays pivotal functions in the regulation of cell proliferation, cell death, apoptosis, and other features of cancerous cells (Wu et al., 2019). Considering its interfering role in different molecular pathways including NF-kB, JNK has binary roles in cancer development/progression (Tournier, 2013). By inhibiting the phosphorylation of glycogen synthase kinase 3 beta (GSK3β), JNK has suppressive effects on the expression of β-catenin (Hu et al., 2009). Ali et al. reported that *L. casei* probiotic supplementation in mice with 1,2-dimethylhydrazine-induced colon cancer significantly reduced the number of aberrant crypt foci compared with that in untreated animals. Furthermore, by upregulating the expression of phosphorylated JNK-1, *L. casei* regulated the expression of β-catenin and phosphorylated GSK3β, leading to significant protective effects against colon cancer (Ali et al., 2019). Sakatani and others have demonstrated that a *L. brevis*-derived polyphosphate significantly promoted the activation of the ERK signaling pathway, expression of cleaved PARP, and the ratio of cleaved PARP/PARP in SW620 colon cancer cells and mice bearing SW620 tumor xenografts. These changes led to increased apoptosis and inhibition of colon cancer growth (Sakatani et al., 2016a). By increasing the level of various inflammatory cytokines including IL-18, TNF-α, and TGF-β1, the NLR family pyrin domain–containing 3 (NLRP3) inflammasome can trigger metastasis in colon and colorectal cancer samples (Shaima’a Hamarsheh, 2020). The results of a recent study demonstrated that probiotic supplementation with the *E. faecalis* strain caused inhibitory effects on the activation of caspase-1 and maturation of IL-1β *in vivo*. Furthermore, *E. faecalis* suppressed the activation of NLRP3 inflammasome, and thereby protected animals from intestinal inflammation in dextran sodium sulfate-induced colitis-associated colorectal cancer (Chung et al., 2019a). Two-week intervention with *L. casei* in 1,2-dimethylhydrazine dihydrochloride-induced colon cancer in mice reduced the occurrence of chemical-induced aberrant crypt foci and the activity of ornithine decarboxylase. As noted previously, by promoting the polyamine metabolism in tumoral cells, ornithine decarboxylase has a pivotal function in the induction of cell proliferation. Hence, suppression of this enzyme *in vivo* diminished colon cancer growth and proliferation (Irecta-Nájera et al., 2017a). Numerous investigations have demonstrated that the expression level of insulin-like growth factor-1 (IGF-1) and IGF-1 receptor (IGF-1R) in colorectal cancer patients is associated with poor prognosis, chemoresistance, and increased invasiveness features (Shiratsuchi et al., 2011; Vigneri et al., 2015). Valadez-Bustos and others demonstrated that probiotic intervention with *B. longum* BAA-999 in the colorectal murine model reduced the expression level and activity of IGF-1/IGF-1R in a considerable manner. Furthermore, after probiotic supplementation, the expression level of insulin-like growth factor-binding protein-3 (IGFBP3) was normalized. Overall, the noted alterations led to reduction in the tumor volume and size (Valadez-Bustos et al., 2019). In a comprehensive *in vivo* investigation, Jacouton and others found that by decreasing the expression grade of IL-22 as a pro-inflammatory cytokine and upregulating the expression of caspase-7, caspase-9, and Bik, probiotic treatment with *L. casei* BL23 had significant anti-proliferative effects in the azoxymethane-induced colorectal cancer model (O'Mahony et al., 2001). Table 3 provides a summary of *in vivo* investigations on the efficacy of probiotics in colorectal cancers.

**TABLE 3:** Probiotics and colon cancer in animal studies.

Probiotic agent	Probiotic concentration	Duration of the study	Effect (s)	Ref.
*Bifidobacterium longum* BAA-999	8.992 × 10^10^ CFU/ml	16 weeks	Regulated IGF-1, IGF-1R, and IGFBP3 protein expressions	Valadez-Bustos et al. (2019)
VSL#3	1.5 × 10^9^ CFU	3 months (5 days weekly)	The level of TNF-α and IL-6 was reduced in colon tissue and tumor load after probiotic intervention	Wang et al. (2018b)
VSL#3	10^9^ CFU daily	18 weeks	Altered the microbial composition	Arthur et al. (2013)
*Lactobacillus plantarum*	1 × 10^9^ CFU/ml	8 months	Reduced β-galactosidase and β-glucuronidase activities. Besides, reduced the number of total coliforms	Čokášová et al. (2012)
*Lactobacillus casei* strain Shirota	2.1 × 10^10^	8, 12, and 25 weeks	Significantly inhibited aberrant crypt foci and colon carcinogenesis	Yamazaki et al. (2000)
*Lactobacillus fermentum* and *Lactobacillus plantarum*	2 × 10^8^ CFU/g and 2 × 10^8^ CFU/g	21 days	Decreased the number of crypts in the mice and the activities of β-galactosidase and β-glucuronidase	Asha and Gayathri, (2012)
VSL#3	1.3 × 10^6^ CFU	44 days	Protected against carcinogenesis through regulating the IL-6/STAT3 signaling pathway	Do et al. (2016)
*Saccharomyces boulardii*	3 × 10^8^ CFU/ml and 6 × 10^8^ CFU/ml	9 weeks	Suppressed HER-2, HER-3, IGF-1R, EGFR-Erk, and EGFR-Akt expression levels and intestinal tumor growth	Chen et al. (2009)
*Lactobacillus delbrueckii subsp. bulgaricus* and *Streptococcus thermophilus*	less than 1 × 10^2^ CFU/ml	5 months	Reduced β-glucuronidase and nitroreductase activity	de Moreno de LeBlanc and Perdigón, (2005)
*Lactobacillus casei* ATCC 393	10^6^ CFU	2 weeks	Showed protective effects against ornithine decarboxylase activities	Irecta-Nájera et al. (2017b)
*Lactobacillus acidophilus* and *Lactobacillus rhamnosus GG*	1 × 10^9^ lactobacilli/0.1 ml	18 weeks	Caused decrease in Bcl-2 and K-ras and increase in Bax and p53 expression levels. Promoted Bax-mediated apoptosis in colon carcinogenesis	Sharaf et al. (2018)
*Lactobacillus rhamnosus* GG MTCC #1408*, Lactobacillus casei* MTCC #1423, and *Lactobacillus plantarum* MTCC #1407	1 × 10^9^ CFU/0.1 ml	7 weeks	Probiotic administration decreased the activity of β-glucosidase	Verma and Shukla, (2013)
*Lactobacillus casei* BL23	5 × 10^9^ CFU/ml	53 days	Decreased the expression of IL-22 while increased the expression of caspase-7, -9, and Bik	Jacouton et al. (2017)
*Lactobacillus salivarius ssp. salivarius* UCC118	NA	16 weeks	Reduced the number of fecal coliform and enterococci levels	O'Mahony et al. (2001)
*Enterococcus faecium* CRL 183	NA	42 weeks	Increased the immune response by promoting the expression of NO, IL-4, IFN-γ, and TNF-α	Sivieri et al. (2008)
*Lactobacillus acidophilus* LaVK2 and *Bifidobacterium bifidum* BbVK3	2 × 10^9^ CFU/g of each strain (20 g)	32 weeks	Probiotics decreased the pre-neoplastic lesions and PCNA expression level	Mohania et al. (2014)
VSL#3	333 × 10^9^ CFU/g	115 days	Promoted angiostatin, VDR, and alkaline sphingomyelinase expression	Appleyard et al. (2011)
*Bifidobacterium longum, Lactobacillus acidophilus,* and *Enterococcus faecalis*	1 × 10^7^ CFU of each	9 weeks	Alleviated colitis through regulating CXCR2 signaling	Song et al. (2018)
*Enterococcus faecalis* KH2	17 mg/kg	2 weeks	Modulated the activity of the NLRP3 inflammasome and ameliorated colitis-associated colorectal cancer	Chung et al. (2019b)
*B. bifidum* (Bla/016P/M) and *Lactobacillus acidophilus*	1 × 10^9^ CFU/g of each strain	10 days before tumor induction and 5 months after it	IFN-γ and IL-10 serum levels and the number of CD4^+^ and CD8^+^ cells were decreased after probiotic administration	Agah et al. (2019b)
*Lactobacillus acidophilus, Lactobacillus rhamnosus,* and *Bifidobacterium bifidum*	0.6 × 10^6^ CFU of each strain	1 week	Reduced the expression of RANTES, eotaxin, p-IKK, and TNF-α while increased IL-10 expression	Mendes et al. (2018)
*Lactobacillus salivarius* Ren	5×10^8^ and 1 × 10^10^ CFU/kg	2 weeks	Prevented carcinogenesis by regulating the intestinal microflora	Zhu et al. (2014)
*Lactobacillus rhamnosus* GG CGMCC 1.2134	1 × 10^9^ CFU/1 ml	25 weeks	β-catenin, Bcl-2, NFkB-p65, COX-2, and TNF-α expression levels were decreased after probiotic intervention	Gamallat et al. (2016)
*Lactobacillus plantarum* AS1	10^9^ CFU/ml	26 weeks	Had antioxidant-induced prevention of colon carcinogenesis	Kumar et al. (2012)
*Lactobacillus casei* Zhang	4 × 10^9^ CFU	NA	Suppressed tumorigenesis through modulating various adiponectin-elevated signaling pathways	Zhang et al. (2017)
*Lactobacillus casei* BL23 and *Lactococcus lactis* MG1363	1 ± 0.4 × 10^9^ CFU/mouse	6 months	Along with the modulation of regulatory T-cells, promoted the expression of IL-6, IL-17, IL-10, and TGF-β	Lenoir et al. (2016)
*Bacillus subtilis*-SKm (KFCC11520P) and *Lactococcus lactis*-GAm (KFCC11510P)	10^6^ CFU/g of *Bacillus subtilis*-SKm and 10^6^ CFU/g of *Lactococcus lactis-*GAm	4 weeks	Probiotics decreased the expression of iNOS, COX-2, and Bcl-2 while increased Bax, p21, and p53 expression levels	Jeong et al. (2012)
VSL#3	333 × 10^9^ CFU/g	2 weeks	Reduced the expression of TNF-α, IL-1β, IL-6, and COX-2 while increased IL-10 expression	Talero et al. (2015)
*Propionibacterium freudenreichii* TL133	2 × 10^10^ CFU/ml	18 days	Increased the induction of apoptosis	Lan et al. (2008)
VSL#3	1.2 × 10^9^ bacteria per day	32 days	Increased the expression of TNF-α, angiostatin, IL-17, and PPAR-γ	Bassaganya-Riera et al. (2012)
*Lactobacillus acidophilus* and *Bifidobacterium animalis subsp. lactis* and both of them	5 × 10^7^ CFU/g and 5 × 10^7^ CFU/g and both strains (2.5 × 10^7^ CFU/g for each strain)	10 weeks	Increased the expression of caspase-3 and decreased the expression of Bcl-2	Lin et al. (2019)
*Lactobacillus acidophilus*	10^10^ CFU/ml	12 weeks	Adenomas have been reported to be decreased after probiotic administration	Urbanska et al. (2009)
*Streptococcus thermophilus* CRL807 and *Lactobacillus delbrueckii* subsp*. bulgaricus* CRL864	NA	5 days	Prevented colitis and carcinogenesis via modulating anti-inflammatory responses	Del Carmen et al. (2016)
*Lactobacillus plantarum* (AdF10) and *Lactobacillus rhamnosus* GG (LGG)	1 × 10^10^ CFU	16 weeks	Regulated COX-2 expression	Walia et al. (2015)
VSL#3	1.3×10^6^ bacteria	8 weeks	Diminished the severity of colitis and tumor growth	Chung et al. (2017)
*Lactobacillus acidophilus*	2 × 10^8^ CFU/ml	1 month	Attenuated COX‐2, iNOS, and c‐Myc expression levels	Deol et al. (2018)
*Lactobacillus plantarum* (AdF10) and *Lactobacillus rhamnosus GG* (LGG)	10^10^ CFU/ml	16 weeks	Had chemopreventive effects	Walia et al. (2018)
*Lactobacillus acidophilus* CL1285*, Lactobacillus casei* LBC80R, and *Lactobacillus rhamnosus* CLR2	At least 50 × 10^9^ CFU/g of strains	12 weeks	Decreased the activity of β-glucosidase and β-glucuronidase along with the reduction in aberrant crypt foci counts	Desrouillères et al. (2015)
*Lactobacillus plantarum* A and *Lactobacillus rhamnosus* b	1 × 10^8^ CFU for 14 consecutive days, then 1 × 10^9^ CFU for 3 weeks	5 weeks	Increased production of IFN-γ and promoted Th1-type CD4^+^ T differentiation	Hu et al. (2015a)
*Streptococcus thermophilus* CRL807*, Streptococcus thermophilus* CRL807, *Streptococcus thermophilus* CRL807, *Lactococcus lactis subsp. cremoris* MG1363, *Lactococcus lactis* subsp. *cremoris* MG1363, and *Lactococcus lactis* subsp*. cremoris* MG1363	1 × 10^10^ CFU/ml	6 months	Exerted anti-tumorigenic properties via increasing antioxidant enzymes and IL-10 expression level	Del Carmen et al. (2017)
*Lactobacillus acidophilus* (NCK 2025)	5 × 10^8^ CFU	4 weeks	Regulated inflammation and prevented colonic polyposis	Khazaie et al. (2012)
*Lactobacillus acidophilus* (Delvo Pro LA-1)*, Lactobacillus rhamnosus* (GG), *Bifidobacterium animalis* (CSCC 1941), and *Streptococcus thermophilus* (DD145)	10^10^ CFU/g	4 weeks	Suppressed DMH-induced colon cancer in rats	McIntosh et al. (1999)
*Bifidobacterium longum*	NA	NA	Exerted anti-proliferative and anti-oxidative properties	Allen et al. (2015)
*Bifidobacterium adolescentis* SPM0212	1 × 10^8^ CFU	3 weeks	Inhibited activity of harmful enzymes and proliferation	Kim et al. (2008)

IGF-1, insulin-like growth factor 1; IGF-1R, insulin-like growth factor 1 receptor; IGFBP3, insulin-like growth factor-binding protein 3; TNF-α, tumor necrosis factor alpha; IL-6, interleukin 6; STAT3, signal transducer and activator of transcription 3; HER-2, human epidermal growth factor receptor 2; HER-3, human epidermal growth factor receptor 3; EGFR, epidermal growth factor receptor; Bcl-2, B-cell lymphoma 2; Bax, Bcl-2–ssociated X; IL-22, interleukin 22; Bik, Bcl-2–interacting killer; IL-4, interleukin 4; IFN-γ, interferon gamma; PCNA, proliferating cell nuclear antigen; CXCR2, CXC chemokine receptor 2; NLRP3, NLR family pyrin domain–containing 3; RANTES, regulated upon activation, normal T cell expressed, and presumably secreted; IL-10, interleukin 10; NF-κB, nuclear factor kappa B; COX-2, cyclooxygenase 2; IL-17, interleukin 17; TGF-β, transforming growth factor beta; iNOS, inducible nitric oxide synthase; IL-1β, interleukin 1 beta; PPAR-γ, peroxisome proliferator-activated receptor gamma.

#### Probiotics and Colon Cancer in In Vitro Studies

As mentioned earlier, caspase-3 is a pro-apoptotic factor and its decreased levels are associated with the shortened survival time in various types of cancers (Vince et al., 2018). *Bacillus coagulans* (*B. coagulans*) Unique IS2 exerted anti-proliferative and pro-apoptotic properties in the COLO 205 human colon cancer cell line. By activating the p-53-mediated apoptotic pathway, treatment with probiotics increased the expression of BAX, activation of caspase-3, cleavage of poly (ADP-ribose) polymerase, and release of cytochrome *C*. Furthermore, *B. coagulans* reduced the mitochondrial membrane potential and Bcl2 expression level (Madempudi and Kalle, 2017). Orlando and others reported that *L. rhamnosus* GG intervention in Caco-2, HT-29, and SW480 colon cancer cell lines upregulated the Bax/Bcl-2 ratio, increasing apoptosis in these cells (Orlando et al., 2016). The cyclin family is a group of cell cycle regulators. Their aberrant expression is associated with tumorigenesis (Wood and Endicott, 2018). Intervention with *L. paracasei* subsp. *paracasei* reduced the expressions of cyclin D1 and cyclin E1 X12 in HT-29 colon cancer cells. In addition, probiotic intervention upregulated the expression of p27 as a cyclin-dependent kinase (CDK) inhibitor (Huang et al., 2016). CKD inhibition represents a potential mechanism for suppressing over proliferation of cancer cells induced by aberrant regulation of the cyclin family (Sánchez-Martínez et al., 2019). PTEN (phosphatase and tensin homolog) has been demonstrated to be a prominent tumor suppressor gene, which plays critical roles in the dephosphorylation of phosphatidylinositol 3,4,5-trisphosphate (PIP3) (Di Cristofano et al., 1998). The additional evidence indicated that downregulation of PTEN is associated with increased tumor growth and survival. Therefore, targeting PTEN inhibitors is one of the most effective means for decreasing the tumor incidence, tumor volume, and tumor growth rate (Lee et al., 2019a). Sambrani et al. demonstrated, in HT-29 colon cancer cells, that treatment with *Saccharomyces cerevisiae* (*S. cerevisiae*) caused a significant upregulation in the expression of PTEN and caspase-3, while the expression levels of Bcl-xL and RelA were markedly decreased after probiotic intervention (Sambrani et al., 2019). *Pichia kudriavzevii* AS-12 treatment showed considerable cytotoxic properties in HT-29 and Caco-2 cells compared with those in normal control cells. In addition, *Pichia kudriavzevii* upregulated the expression of pro-apoptotic agents including Fas-R, caspase-3, -8, and -9, and BAD protein, while the expression of anti-apoptotic Bcl-2 was decreased after yeast probiotic treatment in mentioned cell lines (Saber et al., 2017). *Bacillus polyfermenticus* treatment reduced ErbB2, ErbB3, cyclin D1, and E2F-1 transcription factor in HT-29, DLD-1, and Caco-2 colon cancer cells. These changes led to the suppression of over proliferation of cancerous cells (Ma et al., 2010). In another study, Lee et al. investigated the anti-cancer effects of the *B. adolescentis*-derived butanol extract in Caco-2, HT-29, and SW480 colorectal cell lines. The results showed that the butanol extract significantly promoted the activation of macrophages and upregulated the production of TNF-α and nitric oxide in tumor cells. These changes led to the induction of cytotoxic and anti-proliferative properties against colorectal cancer (Lee et al., 2008). Survivin is an anti-apoptotic agent, which has been reported to be a crucial agent in the inhibition of apoptosis and subsequent tumor growth, proliferation, metastases, and invasiveness in various types of cancer, especially in colorectal cancer (Hernandez et al., 2011). Tiptiri-Kourpeti et al. demonstrated that *L. casei* ATCC 393 administration (10^9^ CFU/ml) in CT26 and HT29 colon carcinoma cells upregulated the expression of the ligand TRAIL, which was induced by TNF-mediated apoptosis. Furthermore, *L. casei* declined the level of survivin expression (Tiptiri-Kourpeti et al., 2016). By significantly decreasing the expression of Bcl-2 and remarkable up-regulation in the expression grade of pro-apoptotic agents Bak and Bax, probiotic intervention with *L. paracasei* K5 showed anti-proliferative effects in Caco-2 cells (Chondrou et al., 2018). In another investigation, Chen et al. reported that various strains of *Lactobacillus* genera in HT-29 colon cancer cells promoted the expression level of the Bax protein, while decreasing the expression of Bcl-2, leading to a notable increase in the Bax/Bcl-2 ratio. Furthermore, the increased lactate dehydrogenase activity and the ensuing degradation of the cell membrane of tumor cells were observed (Chen et al., 2017). A summary of mechanistic *in vitro* investigations on probiotics and colon cancer is summarized in Table 4.

**TABLE 4:** Probiotics and colon cancer (*in vitro*).

Cancer cell line	Probiotic agent	Probiotic concentration	Effect (s)	Ref.
SW620	*Lactobacillus brevis* SBL8803	NA	Via activating the Erk pathway and inhibiting tumor growth	Sakatani et al. (2016b)
SW620	*Lactobacillus delbrueckii*	NA	Through triggering the caspase 3-mediated pathway and decreasing Bcl-2 and caused apoptosis. Besides, MMP-9 was decreased after intervention	Zhou et al. (2014)
SW742	*Bifidobacterium*	NA	Inhibited the growth of cancer cells	Otte et al. (2008)
SW742	*Bifidobacterium and Lactobacillus*	NA	Prevented the development of colorectal cancer	Bahmani et al. (2019)
Colo320 and SW480	*Lactobacillus acidophilus, Escherichia coli Nissle 1917*, and the probiotic mixture VSL#3	1 × 10^6^ CFU/ml	Regulated the expression of COX-2	Otte et al. (2008)
SW480 and HCT-116	*Lactococcus lactis*	NA	Induced apoptosis in human colon cancer cells and increased the ratio of f Bax/Bcl2	Bohlul et al. (2019)
HCT-116	*Lactobacillus fermentum*	NA	*Lactobacillus* cell-free supernatant activated the intrinsic apoptosis pathway	Lee et al. (2019b)
HCT-116	*Lactobacillus plantarum* 27 (NCDC 012), *Lactobacillus casei* (NCDC 297), and *Lactobacillus brevis* (NCDC 021)	NA	Exerted anti-proliferative activities. Inhibited activity of α-glucosidase and α-amylase	Mushtaq et al. (2019)
HCT-116	*Lactobacillus sp., Lactobacillus casei,* and *Lactobacillus rhamnosus GG*	10^9^–10^11^ CFU/ml	Decreased the expression of MMP-9 and increased protein levels of ZO-1	Escamilla et al. (2012)
HCT-116	*Pediococcus pentosaceus* GS4	1.1 × 10^9^ CFU/ml	Downregulated NF-κB and p-Akt signaling pathways	Dubey et al. (2016)
HCT-116, AGS, A549, MCF-7, and HepG2	*Aspergillus sp*	NA	Exhibited anti-tumor properties	Choi et al. (2011)
HT-29, HCT-116, and Caco-2	*Bifidobacterium bifidum* BGN4	NA	Inhibited the growth of cancer cell lines	You et al. (2004)
HT-29	*Lactobacillus casei K11, Lactobacillus casei M5, Lactobacillus casei SB27*, *and Lactobacillus casei × 12*	NA	Cell cycle arrest induced at the G0/G1 phase	Di et al. (2018)
HT-29	*Lactobacillus kefiri* (SGL 13)	5 × 10^8^ CFU/ml	Increased Bax expression and decreased the caspase 3, mutant p53, and IL-8 expression	Brandi et al. (2019)
HT-29	*Enterococcus faecium* YF5	1 × 10^11^ CFU	Inhibited foodborne pathogens	Tan et al. (2013)
HT-29	*Lactobacillus acidophilus* 145 *and Bifidobacterium longum* 913	10^6^–10^8^ and 10^5^ CFU/g	Increased oxidative-induced damage	Oberreuther-Moschner et al. (2004)
Caco-2 and HT-29	*Lactobacillus rhamnosus* MD 14	NA	Showed anti-genotoxic and cytotoxic properties against colon cancer	Sharma et al. (2019)
HT-29	*Lactobacillus casei* 01	10^9^ CFU/ml	Exerted cytotoxic effects	Liu et al. (2011b)
HT-29	*Lactobacillus casei* ATCC 393*, Lactobacillus* plantarum ATCC 14917*,*and *Lactobacillus paracasei* K5	10^9^ CFU/ml	Caused a significant decrease in proliferation of cancer cells in a time- and dose-dependent manner	Mantzourani et al. (2019)
HT-29 and Caco-2	*VSL3*(*Lactobacillus acidophilus, Lactobacillus bulgaricus, Lactobacillus casei, Lactobacillus plantarum, Bifidobacterium breve, Bifidobacterium infantis, Bifidobacterium longum,* and *Streptococcus thermophilus*)	NA	Increased the expression of PPARγ	Ewaschuk et al. (2006)
HT-29 and L-929	*Lactobacillus paracasei* and *Lactobacillus brevis*	NA	Induced apoptosis in cancer cells	Mojibi et al. (2019)
HT-29	*Lactobacillus acidophilus* 606	NA	Exerted anti-tumorigenic properties by inducing the expression of Beclin-1, GRP78, Bcl-2, and Bak	Kim et al. (2010)
HT-29 and HCT-116	*Lactobacillus plantarum*	NA	Increased the activity of caspase-3 and suppressed the Wnt/β-catenin signaling pathway. Therefore, reversed chemoresistance and enhanced the therapeutic effect of 5-FU in colon cancer	Mirzaei et al. (2016)
HT-29 and HCT-116	*Lactobacillus* spp	3 × 10^8^ CFU/ml	Down-regulated expression of IL-1β and TNF-α.cfos and cjun transcripts were significantly upregulated after probiotic intervention	Shyu et al. (2014)
HT-29	*Lactobacillus paracasei* subsp. *paracasei* M5L	10^9^ CFU/ml	Via generating ROS production, inducing cell cycle arrest, and calreticulin translocation	Hu et al. (2015b)
HT-29	*Leuconostoc mesenteroides*	NA	By regulating MAPK1, Bax, and caspase 3 and downregulation of Akt, NF-Kb, and Bcl-XL promoted apoptosis. Besides, suppressed the expression of miRNA-21 and miRNA-200b	Zununi Vahed et al. (2017)
HT-29, Caco2, and HeLa	*Propionibacterium acidipropionici* strain CNRZ80*, Propionibacterium freudenreichii subsp. freudenreichii* strain ITG18, and *Propionibacterium freudenreichii subsp. shermanii* strain SI41	NA	Via short-chain fatty acids acting on the mitochondria, caused apoptosis in cancer cells	Jan et al. (2002)
HT-29 and HCT-116	*Propionibacterium freudenreichii*	NA	Induced apoptosis by increasing pro-apoptotic gene expression (TRAIL-R2/DR5) and decreasing FLIP and XIAP.	Cousin et al. (2016)
Caco-2	*Bifidobacterium animalis subsp. lactis* DSM10140, *Bifidobacterium longum* subsp. *longum* DSM20097, and *Bifidobacterium breve* DSM20213	>5.0 logs CFU/g	Caused remarkable cytotoxic activities	Ayyash et al. (2018)
Caco-2	*Lactobacillus rhamnosus* and *Bifidobacterium lactis*	10^8^ CFU/ml	Induced FAS-independent apoptosis and increased BAX translocation and release of cytochrome *c* and cleavage of caspase-3 and -9	Altonsy et al. (2010)
Caco-2 and HT-29	*Lactobacillus plantarum* A7 and *Lactobacillus rhamnosus* GG	NA	Decreased the growth rate of cancer cells	Sadeghi-Aliabadi et al. (2014)
Caco-2	*Escherichia coli* Nissle 1917	25 × 10^7^ CFU	Decreased ROS generation	Wang et al. (2015)
Caco-2	*Lactobacillus plantarum*	NA	Upregulated the mRNA expression of HBD-2 and modulated the TLR-2 and IL-23 expression	Paolillo et al. (2009)
Caco-2	*Lactobacillus paracasei*	10^8^ CFU/ml	Inhibited the mRNA expressions of CXCR4	Nozari et al. (2019)
Caco-2	*Pediococcus pentosaceus* FP3*, Lactobacillus salivarius* FP25*, Lactobacillus salivarius* FP35*,* and *Enterococcus faecium* FP51	NA	Triggered the biosynthesis of short-chain fatty acids	Thirabunyanon and Hongwittayakorn, (2013)
Caco-2 and CLS	*Enterococcus faecium* RM11 and *Lactobacillus fermentum* RM28	NA	Triggered anti-proliferative activities in colon cancer cells	Thirabunyanon et al. (2009)
Caco2, SKCO-1, SW620, and IEC-18	*Lactobacillus casei* ATCC334	NA	Suppressed colon cancer progression via affecting the JNK pathway	Konishi et al. (2016)
DLD-1	*Lactobacillus rhamnosus* strain GG	10^8^ CFU/ml	Exerted anti-proliferative effects	Orlando et al. (2009)
DLD-1	*Lactobacillus rhamnosus (LR)* KCTC 12202BP	NA	Inhibited cell proliferation through affecting the p53-p21-cyclin B1/Cdk1 signaling pathway	An et al. (2019)
TC-1	*Lactobacillus casei* BL23*, Lactococcus lactis* MG1363, and *Lactococcus lactis* NZ9000	1 × 10^9^ CFU of each strain or recombinant	Probiotic strain *Lactobacillus casei* BL23 caused IL-2-mediated anti-tumoral properties	Jacouton et al. (2018)
CT-26	*Lactobacillus casei* variety *rhamnosus* (Lcr35)	1 × 10^3–7^ CFU of the probiotics	Downregulated the expression of TNF-α and IL-6	Chang et al. (2018)
CT-26	*Lactobacillus acidophilus* NCFM	1 × 10^8^ CFU	Suppressed tumor growth in intestinal tissue	Chen et al. (2012)
MCF-7, HT-29, HeLa, HepG2, HL60, K562, and MCF-10A	*Lactobacillus plantarum* strains	NA	Caused anti-proliferative and pro-apoptotic effects against malignant cancer cells	Chuah et al. (2019)
LS513	*Lactobacillus acidophilus CL1285* and *Lactobacillus casei* LBC80R	10^8^ CFU/ml	Via upregulating the caspase-3 protein and enhanced the pro-apoptotic capacity of the 5-FU.	Baldwin et al. (2010)

### Probiotics and Other Gastrointestinal Cancer

The mitogen-activated protein kinase (MAPK) signaling pathway has crucial roles in the induction of intracellular responses from extracellular signals in cells. Aberrant regulation of this pathway leads to numerous homeostatic and pathologic sequels, such as cancer (Chapnick et al., 2011). In the KB oral cancer cell line, probiotic intervention with *L. plantarum* reduced the expression of MAPK and caused significant upregulation in the expression of PTEN signaling transduction (Asoudeh-Fard et al., 2017). Zhang and others evaluated the properties of *Lactobacillus salivarius* (*L. salivarius*) REN supplementation in an animal model of 4-nitroquinoline-1-oxide-induced oral cancer. By decreasing the expression level of COX-2 and proliferating cell nuclear antigen (PCNA), *L. salivarius* intervention had significant inhibitory effects on tumor growth of oral cancer (Zhang et al., 2013). Another study demonstrated that *Acetobacter syzygii* and *L. acidophilus* (PTCC 1643) probiotic strains caused significant cytotoxicity and inhibitory effects against the KB cancer cell line (Aghazadeh et al., 2017). Barrett’s esophagus is a pathological condition in which the lining of the distal esophagus is damaged due to the exposure of the esophagus to stomach acid. In this situation, squamous epithelium of the esophagus is replaced by columnar epithelium (Spechler, 2013). Barrett’s esophagus plays a critical role in the induction of esophageal cancer and acts as an important risk factor for development of esophageal cancer (Conteduca et al., 2012). Mozaffari Namin et al. reported that *B. longum* and *L. acidophilus* treatment of Barrett’s esophagus cell lines downregulated the expression of CDX1 (caudal type homeobox 1), COX-2, TNF-α, and p53, while the expression level of IL-18 was enhanced after intervention of both probiotic strains (Mozaffari Namin et al., 2015). Table 5 provides a summary on the effectiveness of probiotics for oral, esophageal, and pancreatic cancer.

**TABLE 5:** Probiotics and other gastrointestinal cancers.

Cancer	Probiotic agent	Probiotic concentration	Duration of the study	Effect (s)	Model	Sample (n)	Ref.
Oral cancer	*Lactobacillus plantarum*	NA	2, 6, and 24 h	Displayed apoptosis effects via upregulating PTEN and downregulating MAPK signaling pathways	*In vitro*	NA	Asoudeh-Fard et al. (2017)
Oral cancer	*Lactobacillus salivarius REN*	5 × 10^10^ CFU/kg per day	32 weeks	Inhibited rat oral cancer progression through regulating the expression of COX-2 and PCNA.	*In vivo*	NA	Zhang et al. (2013)
Oral cancer	*Acetobacter* *syzygii* and *Lactobacillus acidophilus* (PTCC 1643)	*Acetobacter* *syzygii*: 60 μg/ml and *Lactobacillus acidophilus*: 10 μg/ml	24 h	Exhibited cytotoxicity against cancer cell lines	*In vitro*	NA	Aghazadeh et al. (2017)
Barrett’s esophagus	*Bifidobacterium longum* and *Lactobacillus acidophilus*	3 × 10^7^ bacteria or microbes/ml	1, 3, 5, and 7 h	Increased the expression of IL-18 while decreased the expression of CDX1, COX-2, and TNF-α	*In vitro*	NA	Mozaffari Namin et al. (2015)

PTEN, phosphatase and tensin homolog; MAPK, mitogen-activated protein kinase; COX-2, cyclooxygenase 2; PCNA, proliferating cell nuclear antigen; IL-18, interleukin 18; CDX1, caudal type homeobox 1; COX-2, cyclooxygenase 2; TNF-α, tumor necrosis factor alpha.

## Conclusion

Owing to their effects on different aspects of host health, probiotics have been demonstrated to be important tools in clinical medicine. Various investigations using a plethora of experimental models, including *in vitro*, animal models, and human clinical studies, have shown that by inducing anti-carcinogenic properties, anti-mutagenic effects, producing short-chain fatty acids, activating the immune system of the hosts, inhibiting the bacteria-induced conversion of pro-carcinogens to carcinogens, and reducing intestinal pH (which results in reduced microbial activity), probiotics can assist in the prevention and treatment of gastrointestinal cancers. Nonetheless, to date, the benefits of probiotic strains as bio-therapeutic agents have not been adequately investigated against GI cancers. Moreover, the clinical efficacy of probiotics, especially on mortality, remains largely unexplored. Hence, more clinical studies with adequate follow-up durations are needed to obtain a clearer understanding on the potential utility of various strains and optimal doses for the administration of probiotics as pharmacological tools to combat GI cancers.
